# Peptide inhibition of the SETD6 methyltransferase catalytic activity

**DOI:** 10.18632/oncotarget.23591

**Published:** 2017-12-21

**Authors:** Michal Feldman, Dan Levy

**Affiliations:** ^1^ The Shraga Segal Department of Microbiology, Immunology and Genetics, National Institute for Biotechnology in the Negev, Ben-Gurion University of the Negev, Be'er-Sheva 84105, Israel; ^2^ National Institute for Biotechnology in the Negev, Ben-Gurion University of the Negev, Be'er-Sheva 84105, Israel

**Keywords:** Lysine methylation, SETD6

## Abstract

A large body of evidence accumulating in the past few years indicates the physiological significance of non-histone proteins lysine methylation, catalyzed by protein lysine methyl transferases (PKMTs). Dysregulation of these enzymes was shown to contribute to the development and progression of numerous diseases. SETD6 lysine methylatransferase was recently shown to participate in essential cellular processes, such as the NFkB pathway, oxidative stress and also the Wnt signaling cascade. In order to test the effect of blocking SETD6 catalytic activity, we used the peptide inhibition method, which is considered highly specific and can potentially target almost any protein. We designed a 15 amino acids peptide based on the sequence of the RelA protein (residues 302-316), containing the lysine that is methylated by SETD6. To enable cellular intake, the designed peptide was fused to a cell penetrating peptide (CPP) vp22. The vp22-RelA302-316 peptide showed direct and specific interaction with SETD6 *in vitro*. This interaction was shown to inhibit SETD6 methyltransferase activity. SETD6 catalytic blockage by the peptide was also observed in cells upon treatment with the vp22-RelA302-316, resulting in induced cellular migration and proliferation. This new insight into the activity of a methylation inhibitory peptide could represent a milestone in the development of therapeutic tools, which can be of use in physiological cases where administration of cell proliferation is required.

## INTRODUCTION

Post-translational modifications (PTMs) contribute significantly to the structural and functional diversity of the proteins. These modifications confer complexity to the eukaryotic proteomes that is several orders of magnitude greater than the coding capacity of the genome [1]. Due to their pivotal role in physiological processes, in-depth understanding of protein PTMs is important not only for gaining a perception of a wide array of cellular functions but also towards developing drug therapies for many life-threatening diseases [2].

In comparison to intensively studied PTMs such as phosphorylation, acetylation and ubiquitination [3], the methylation of lysine residues and their functional physiological consequences in tumorigenesis is still a developing field. A lysine residue in a given protein can be mono, di or tri-methylated by protein lysine methyltransferases (PKMTs). PKMTs are a family of approximately 50 proteins, all containing the catalytic SET domain [4, 5]. Growing amount of studies have shown that dysregulation of PKMTs has substantial roles in tumorigenesis [4], and therefore efforts have been put in elucidating substrate specificity of these enzymes.

One of the recently studied PKMTs is SETD6, which was shown to participate in several essential signaling pathways, such as NFƙB [6], WNT [7], oxidative stress response [8] and nuclear hormone receptor signaling [9]. The growing list of SETD6 substrates strongly establishes its versatile role in vital cellular processes. Its versatility is characterized by the dependency on cell types and origins, and in different events and stages in the cellular life course, for example differentiation, cell cycle, DNA damage, tumorigenesis and many more. Thus, understanding the consequences of SETD6 contribution to these pathways would represent a hallmark of its investigation.

One of the ways to control the activity (rather than expression) of a desired protein target is the use of specifically designed peptides. Peptides are a promising tool for targeting proteins since they have several advantages: they are easily synthesized, they have a high target specificity and selectivity and their cytotoxicity is considered low. For that reason therapeutic peptides represent a novel approach to treat many diseases including cancer [10]. Indeed, peptides are an ongoing studied tool for the therapeutic of numerous diseases, such as cancer [10], neurodegenerative diseases [11] and diabetes mellitus [12] and they have also been investigated as a potential antimicrobial [13, 14] or antiviral [15] treatment.

Herein we present a study in which a 15 amino acid synthetic peptide, designed based on the RelA sequence and containing the lysine residue known to be methylated by SETD6 [6], has a remarkable inhibitory effect on the methyltransferase activity of SETD6. We show that this peptide, when conjugated to the vp22 cell penetrating peptide [16, 17], is able to cross the cell membrane, interact with SETD6 and inhibit its catalytic activity, leading to extensive migration and proliferation. These results are of high value in deciphering the specific cellular role of SETD6 and might also be of use in developing therapeutic tools for the regulation of cell proliferation.

## RESULTS

### Over-expressed RelA peptide binds SETD6 and inhibits its methyltransferase activity

**Figure 1:**
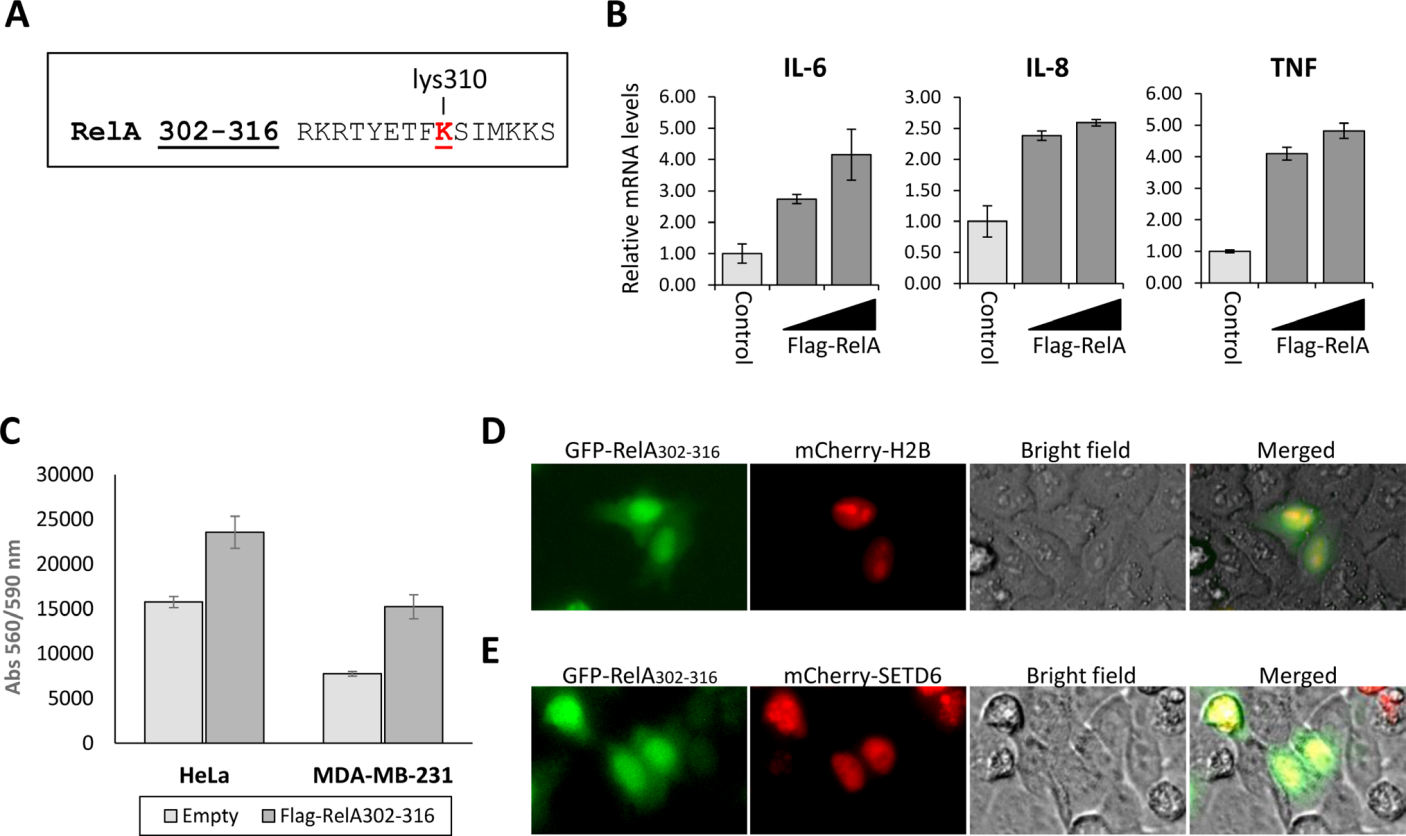
Over-expressed RelA peptide co-localizes with SETD6 and inhibits its catalytic activity in cells (**A**) The 15 amino acids (302-316) sequence of the RelA peptide. Methylated lysine residue (K310) is labeled in red. (**B**) MCF7 cells were transfected with increasing amounts of the Flag-RelA302-316 plasmid. 24 hours post-transfection, cells were harvested, RNA was extracted and used for a qPCR analysis of the indicated genes. mRNA levels were normalized to GAPDH and to control cells. Error bars represent triplicates. (**C**) HeLa or MDA-MB-231 cells were plated at 15,000 cells/well and transfected either with Flag-RelA302-316 or an empty plasmid. 48 hours after transfection viability was measured using the PrestoBlue reagent. Standard deviation represents quadruplicates of two independent experiments. HeLa cells were transfected with the indicated combinations of the GFP-RelA302-316, mCherry-H2B (**D**) and mCherry-SETD6 (**E**). 24 hours after transfection cells were visualized by fluorescent microscopy.

The structure of SETD6 in complex with the RelA peptide containing the methylation site in the presence of S-adenosyl-methionine (SAM) was solved [20]. This short peptide contains residues 302-316 (Figure 1A) of the RelA protein, among them is lysine310, known to be methylated by SETD6. SETD6 monomethylation of RelA at K310 attenuates NF-κB signaling [6], and the fact that this peptide binds to SETD6 catalytic pocket, directed us to speculate that over-expressing this peptide in cells might lead to catalytic inactivation of SETD6 and to the activation of NF-kB target genes [6]. To test this hypothesis, increasing amounts of the Flag-tagged RelA peptide were overexpressed in HeLa cells, followed by qPCR analysis to determine the expression of NF-κB target genes (Figure 1B). Indeed, mRNA levels of the IL6, IL8 and TNF genes significantly increased in a dose dependent manner of ectopically expressed peptide, in comparison to control cells. These results indicate that the expression of RelA (spanning amino acids 302-316) inhibits the transcription activity of RelA in cells (Figure 1B), which is consistent with previous findings [6]. Since knockdown of SETD6 was shown to lead to accelerated cell proliferation rates [6], we next tested proliferation of HeLa and MDA-MB-231 cells upon RelA302-316 over expression (Figure 1C). Cell counts were quantified using the PrestoBlue reagent 48 hours post-transfection. Ectopic expression of the flag-tagged RelA302-316 peptide clearly caused extensive proliferation of both cell lines, confirming the catalytic inhibition of SETD6 by the RelA302-316 peptide. We next aimed at testing the cellular localization of the peptide. To address this question, we generated a GFP- RelA302-316 fusion and co-transfected it in HeLa cells with mCherry-H2B or mCherry-SETD6, both known to localize to the nucleus [6, 21]. GFP- RelA302-316 was observed throughout the cells, however mainly in the nucleus (Figure 1D), where it co-localized with mCherry-SETD6 (Figure 1E). Our data indicates that the over-expressed RelA302-316 co-localizes with SETD6 in cells, where it inhibits the transcription activation of RelA target genes and leads to accelerated cellular migration and proliferation.

### The synthetic vp22-RelA peptide binds SETD6 *in vitro*

**Figure 2:**
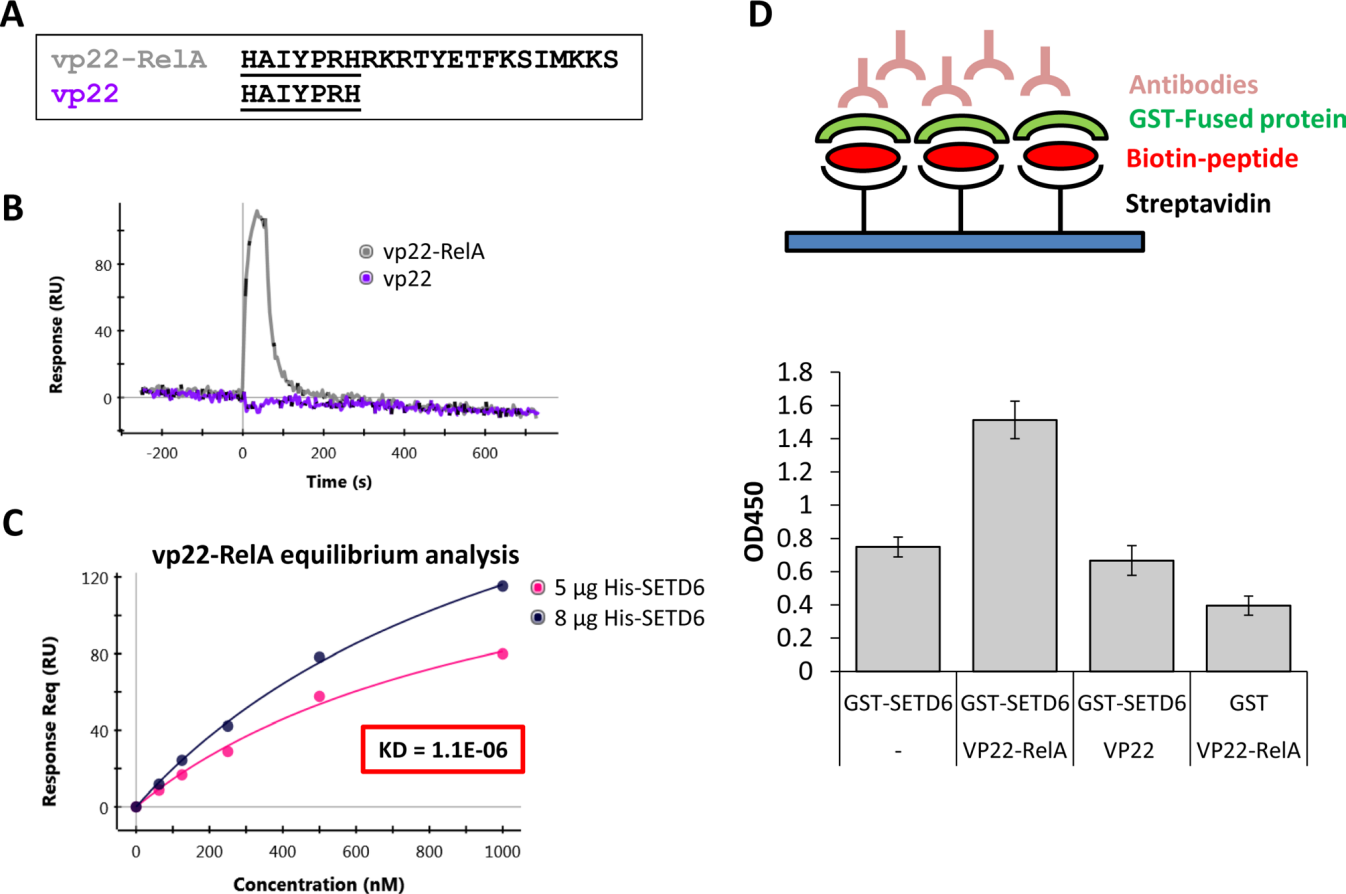
Synthetic vp22-RelA peptide interacts specifically with SETD6 *in vitro* (**A**) Sequence of vp22-RelA and vp22 peptides. (**B**) 1 μM of the vp22-RelA and vp22 peptides (analytes) were allowed to flow over the GLM chip bound His-SETD6. Binding was analyzed using SPR spectroscopy with the ProteOn XPR36 system. (**C**) The dissociation constant (KD) was determined from the sensorgram of the equilibrium binding phase calculated by allowing 5 different concentrations of the vp22-RelA analyte to flow over the chip bound His-SETD6. (**D**) Upper panel - Illustration of the ELISA sandwich experiment performed in the lower panel. ELISA-based analysis of the interaction between recombinant GST-SETD6 and the biotinylated peptides. The 96-well plate was coated with 100 μg streptavidin to which 20 μM biotinylated peptides were bound. Then peptides were covered with 0.5 μg GST-SETD6 or GST. Signal detection was obtained using anti-GST antibody followed by secondary HRP-conjugated antibody (error bars, s.e.m.).

Based on the amino-acid sequence of the RelA302-316 peptide, we designed peptides fused to cell penetrating peptides (CPPs)-signals that facilitate cellular intake [22]. Two different CPPs were tested: vp22 (derived from the herpes virus structural protein VP22) [16, 17] and AntP (the third helix of the homeotic protein of *Drosophila melanogaster* Antennapedia) [23] (Figure 2A and Supplementary Figure 1A, respectively). We next determined if there is a direct interaction between SETD6 and the two synthetic peptides, vp22-RelA and Antp-RelA. Surface-plasmon-resonance (SPR) spectroscopy using the ProteON XPR system, revealed that vp22-RelA interacts with His-SETD6 while the vp22 sequence by itself did not (Figure 2B), implying that the RelA302-316 sequence confers the ability to bind SETD6. In contrast, both the Antp-RelA and the AntP peptides bound His-SETD6 (Supplementary Figure 1B), indicating lack of specificity. Using an equilibrium analysis, we calculated a Kd of 1.1E-06 for the interaction between His-SETD6 and the vp22-RelA peptide (Figure 2C). We further validated this interaction using ELISA test (Figure 2D). Consistent with the SPR results, the vp22-RelA peptide, and not the vp22 peptide alone was observed to interact with GST-SETD6 (Figure 2D). In addition, both the Antp-RelA and Antp peptides interacted with GST-SETD6 (Figure S1C), supporting the results obtained from the SPR analysis. Our data indicates that vp22-RelA specifically binds SETD6 *in vitro*.

### The vp22-RelA peptide penetrates cells and co-localizes with SETD6

**Figure 3:**
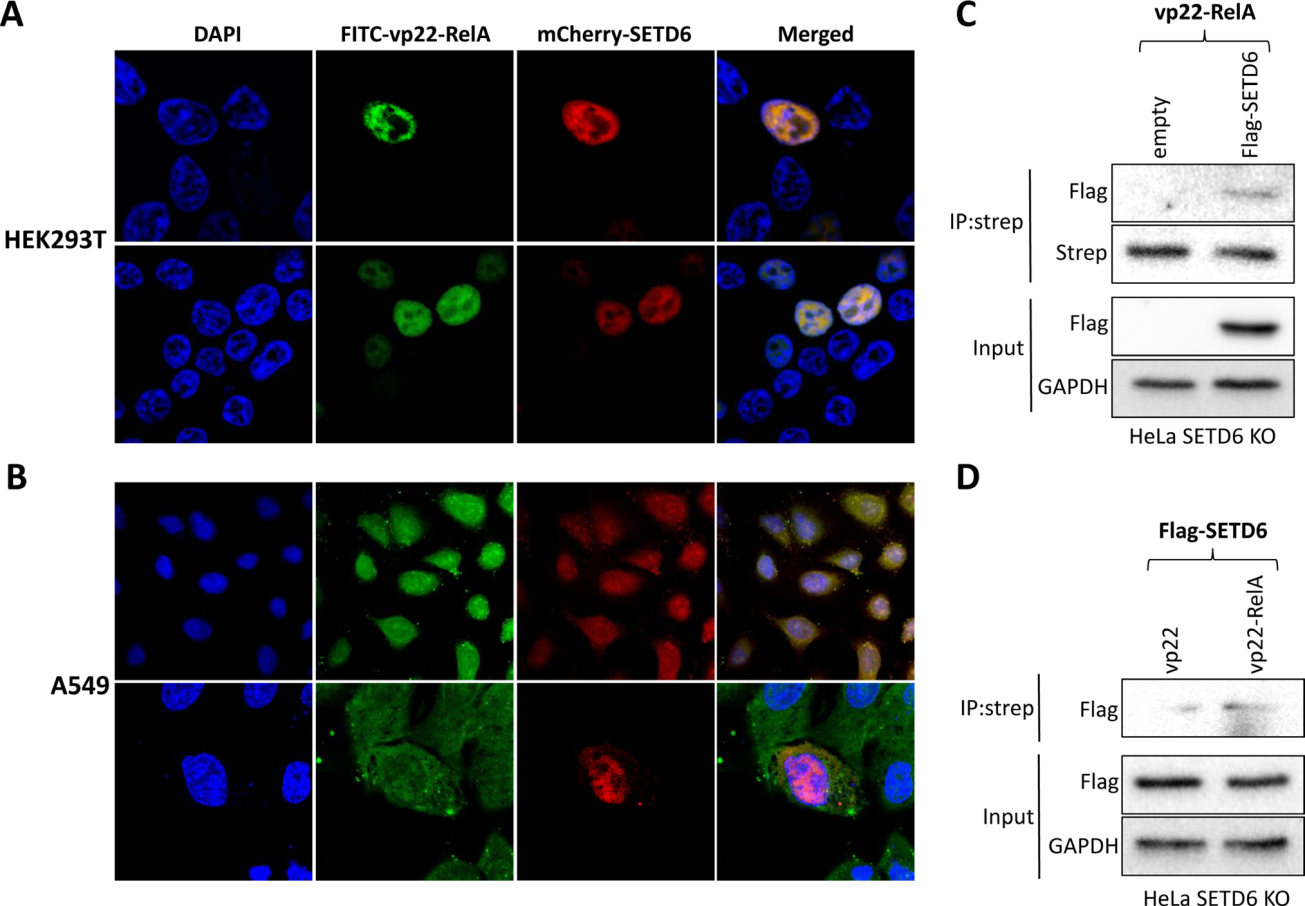
The vp22-RelA peptide penetrates cells and interacts with SETD6 FITC labeled vp22-RelA peptide was introduced into HEK293T (**A**) or A549 (**B**) cells transfected with mCherry-SETD6. Cells were fixed, mounted with DAPI containing mounting solution and visualized using confocal microscopy. Lysates of SETD6 KO HeLa cells stably expressing an empty plasmid or the Flag-SETD6 plasmid were immunoprecipitated with biotin-labeled vp22-RelA302-316 (**C**), or the stably expressing Flag-SETD6 HeLa KO SETD6 cells were immunoprecipitated with biotin-labeled vp22 or vp22-RelA302-316 (**D**) and blotted with Flag antibody to test Flag-SETD6 pull-down. Levels of Flag-SETD6 and GAPDH (loading control) are shown in the lower panels of (C and D).

We next tested the ability of the synthetic vp22-RelA peptide to cross the cell membrane and penetrate into the cytosol and cellular organelles. The vp22 sequence is considered a naturally derived CPP that can mediate potent internalization into the cytoplasm and cell nucleus with no cell specificity [24]. As shown in Figure 3, FITC-tagged vp22-RelA peptide could penetrate both HEK293T (Figure 3A) and A549 (Figure 3B) cells. In addition, expression of mCherry-SETD6 in these cells revealed a strong co-localization in cells of FITC-tagged vp22-RelA peptide and SETD6. Interestingly, in HEK293T cells, the co-localization seemed to take place only in the nucleus, whereas in A549 cells the interaction was observed throughout the cell - both cytosol and nucleus. Translocation of the vp22-RelA peptide across the cell membrane was also detected when the peptide was biotin labeled (Figure S2). The observed colocalization was further supported by SETD6 pull-down from cell lysates by the vp22-RelA302-316 peptide only in HeLa cells expressing Flag-SETD6 (Figure 3C) and not by the vp22 peptide (Figure 3D). Altogether, our data demonstrate that the vp22-RelA peptide internalizes cells and interacts with SETD6.

### The synthetic vp22-RelA peptide inhibits SETD6 catalytic activity

**Figure 4:**
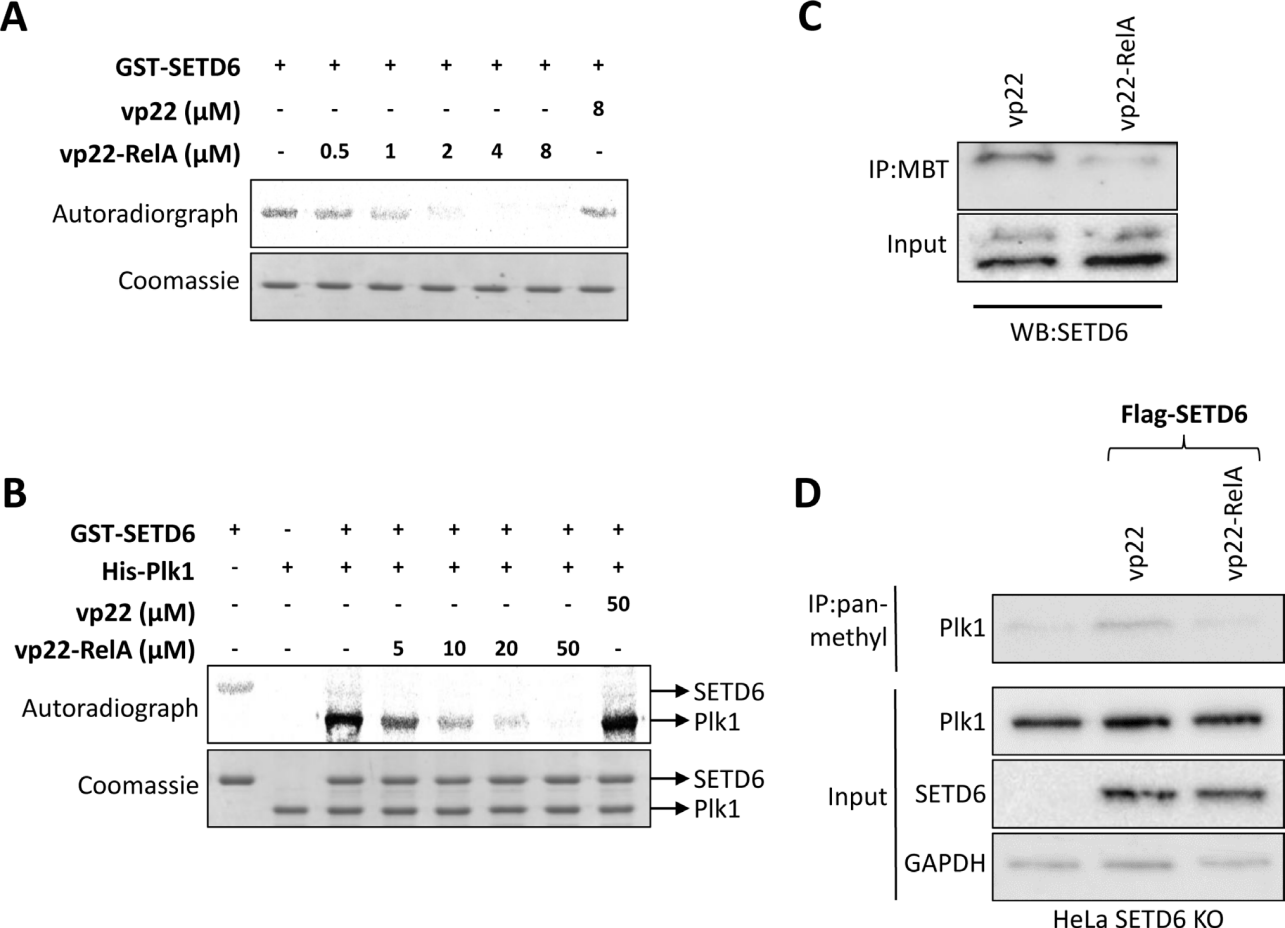
The vp22-RelA peptide blocks SETD6 catalytic activity *in vitro* and in cells (A+B) *In vitro* methylation assay in the presence of ^3^H-labeled S-adenosyl methionine testing GST-SETD6 auto-methylation activity (**A**) or methylation of recombinant His-Plk1 by GST-SETD6 (**B**) while introducing increasing amounts of the vp22-RelA peptide. The vp22 peptide was used as negative control. Coomassie stain of the recombinant proteins used in the reactions is shown on the bottom of each experiment. (**C**) Methylated proteins were pulled-down from cellular extracts of HEK293T cells with His-MBT to detect differences in auto-methylation signal of endogenous SETD6 upon treatment with the vp22-RelA peptide. As negative control, pull-down was also performed with HEK293T cells treated with the vp22 peptide. (**D**) HeLa SETD6 KO cells stably expressing an empty plasmid or the Flag-SETD6 plasmid were treated or not with 40 uM of the vp22 or vp22-RelA302-316 peptides in serum free medium for 4 h, after which cells were washed and fresh complete medium was added overnight. Extracts were immunoprecipitated using pan-methyl antibody and blotted with Plk1 antibody. Endogenous levels of Plk1, SETD6 and GAPDH (loading control) are shown in the lower panel.

Having demonstrated that vp22-RelA specifically binds SETD6, we next continued to assess the hypothesis that the interaction between vp22-RelA and SETD6 inhibits the catalytic activity of SETD6. To this end, we measured the auto-methylation activity of recombinant GST-SETD6 by adding increasing amounts of the vp22-RelA. We observed a significant reduction of SETD6 auto-methylation signal at 0.5 μM peptide and complete abrogation of the signal at 2 μM of the peptide (Figure 4A). This result indicates a strong inactivation of SETD6 auto-methylation activity by the vp22-RelA peptide, and not the vp22 peptide alone (Figure 4A, right lane). Notably, the concentration range of the peptide required for the inhibition was consistent with the SPR results (Figure 2C). Furthermore, we observed that increasing amounts of the vp22-RelA peptide but not vp22 alone, inhibit the methylation of Plk1, a previously identified SETD6 substrate [25] (Figure 4B). Interestingly, a higher dose of the peptide was required for inhibiting Plk1 methylation in comparison to the dose required for complete inhibition of the SETD6 auto-methylation signal. A significant decrease in endogenous SETD6 auto-methylation signal was detected in HEK293T cells using the MBT pull down approach (Figure 4C) which serves as an affinity reagent that specifically binds lysine methylated proteins [18, 19]. This reduction reflects the catalytic inactivation of SETD6 by the vp22-RelA peptide in cells. As expected, the methylation of endogenous Plk1 increased after over-expression of Flag-SETD6 in HeLA SETD6 KO cells in the presence of the vp22 peptide and was dramatically inhibited after treatment with the vp22-RelA peptide (Figure 4D). Our data indicates that the vp22-RelA synthetic peptide specifically inhibits the catalytic activity of SETD6 *in vitro* and in cells.

### vp22-RelA treated cells show faster migration and proliferation

**Figure 5:**
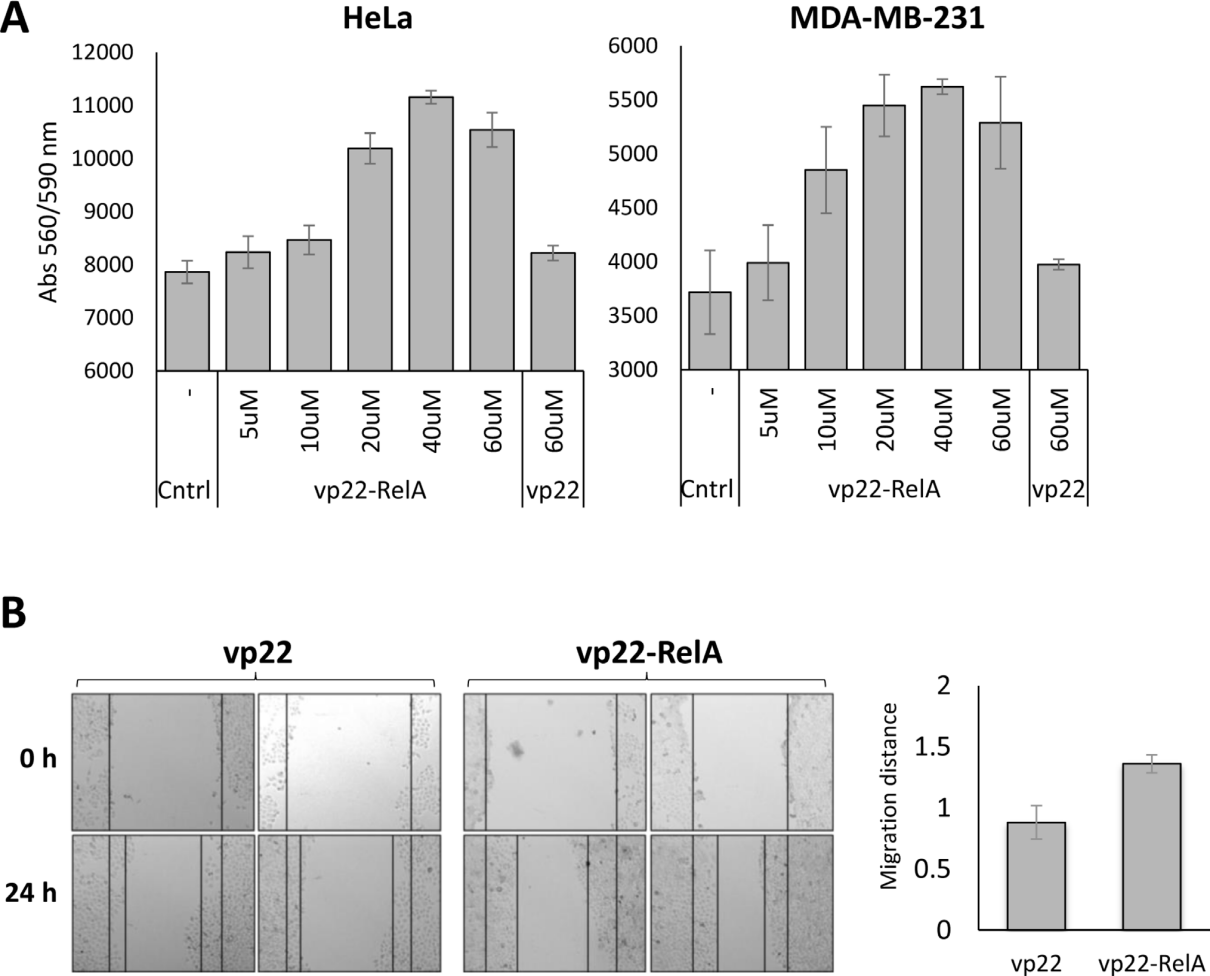
Cells treated with the vp22-RelA peptide show induced proliferation (**A**) HeLa or MDA-MB-231 cells were plated at 20,000 cells/well and treated with increasing amounts of the vp22-RelA peptide in serum free medium for 4 h, after which cells were washed and fresh complete medium was added overnight. The vp22 peptide was used as negative control. After overnight incubation viability was measured using the PrestoBlue reagent. Standard deviation represents triplicates of a representative experiment out of three, all showing similar results. (**B**) HeLa Cells were plated at 25,000 cells/well and supplemented with 40 uM of the vp22 or vp22-RelA peptides in serum free medium for 4 h, after which cells were washed and fresh complete medium was added overnight. A scratch was performed across the center of the well, and pictures were taken. 24 h after which pictures were taken again. On the right - quantitation of the migration distance calculated using ImageJ. Results were normalized to cells treated with the negative control peptide vp22. Standard errors represent six repeats of each condition.

We next sought to understand the functional cellular consequence of SETD6 catalytic inactivation by the peptide. Since depletion of SETD6 is correlated with cell proliferation and tumorigenesis [9, 26–28], we tested the effect of vp22-RelA peptide on related cellular phenotypes. We first examined proliferation of HeLa and MDA-MB-231 cells treated with increasing amounts of the vp22-RelA peptide. Both HeLa and MDA-MB-231 cells showed an increase in cells number 24 hours after peptide treatment in a dose dependent manner using the PrestoBlue reagent, compared to vp22 control and untreated cells (Figure 5A). Consistent with these results, wound-healing assay showed that HeLa cells treated with the vp22-RelA peptide and not with the vp22 peptide migrated faster (Figure 5B, quantified on the right), indicating higher migration capabilities upon SETD6 inhibition following vp22-RelA treatment. Our data implies that consistent with previous observations, the inhibition of SETD6 catalytic activity by the synthetic peptide leads to accelerated proliferation.

## DISCUSSION

PKMTs are rising key players in various cellular processes. Thus, modulation of PKMTs activity represents an attractive target for drug design and compounds that manipulate PKMTs cellular activity are of enormous therapeutic potential. The use of peptides, which copy natural motifs that specifically influence methyltransferase activity and intracellular interactions with its partners, may be a promising approach for selective inhibition of PKMTs. Here we show the novel development of an epigenetic inhibitory peptide which was designed to inhibit the catalytic activity of the SETD6 lysine methyltransferase. We demonstrate that the synthetic peptide binds SETD6 and inhibits its enzymatic activity *in vitro* and in cells. SETD6 inhibition yielded with increasing cellular migration and proliferation.

An inhibitory effect on kinase activity by a peptide has been described before [29]. The PRI peptide, containing a conserved double-stranded RNA binding motif found in PKR fused to a membrane-translocating hydrophobic sequence, was shown to inhibit kinase activity of the double-stranded RNA dependent protein kinase (PKR), a negative regulator of cell proliferation. Inhibition of PKR by the PRI peptide resulted in increased cell density [29]. Another example of a PTM inhibited by a specific peptide was reported in a publication describing the HIV-TAT sequence linked permeable peptide inhibitors of the c-Jun NH_2_-terminal kinase (JNK) signaling pathway [30]. The inhibitory peptides were found to confer cells with full protection against IL-1β induced apoptosis by blocking activation of the transcription factor c-Jun by JNK [30].

Since peptides hold the substantial potential of specific targeting, efforts have been invested in their elaboration, in terms of affinity, turnover and stability [31]. Although the phenotypic outcome of SETD6 substrate methylation inhibition was significant, we believe that such biophysical improvements of vp22-RelA could ameliorate its efficiency to allow the use of lower doses. An attractive approach to improve peptide efficiency is a K-to-M substitution. K-to-M substitutions were shown to represent a mechanism to alter epigenetic states in a variety of pathologies through the inhibition of SET domain enzymes, a conclusion made after the identification of the this mutation in oncohistones linked to numerous cancer types [32–34]

The mechanism through which the vp22-RelA peptide functions in cells is yet to be unraveled, however its ability to induce proliferation implies that SETD6 is involved in cell cycle. Modulation of cell cycle by peptides is not a new concept. The p21^WAF1^ mimicking peptide was found to block phosphorylation of the retinoblastoma protein (pRb) to induce a potent G1/S growth arrest [35]. However, therapeutic uses of a proliferation inducing peptide are conceptually less attractive than a proliferation inhibiting peptide. Nevertheless, such treatment could be of use in cases where regulation of cellular proliferation is required, for example in sustaining the proliferation of cells otherwise difficult to propagate, such as hematopoietic stem cells, nerve and muscle cells and also for promoting wound healing. In addition, such peptide activity might be useful after medical treatments in which cellular resuscitation is necessary, such as chemotherapy.

## MATERIALS AND METHODS

### Cell lines, transfection and treatments

HeLa cells, human embryonic kidney cells (HEK293T), A549 cells and MDA-MB-231 cells were maintained in Dulbecco's modified Eagle's medium (Sigma) with 10% fetal bovine serum (Gibco), 2mg/ml L-glutamine (Sigma), penicillin-streptomycin (Sigma), and non-essential amino acids (Sigma). Cells were cultured at 37°C in humidified incubator with 5% CO_2_. For cell transfection, cells were transfected using Mirus *Trans*IT^®^-LT1 reagent, according to the manufacturer's instructions. Transfection was performed for 24 h before harvesting, counting, or immunofluorescence. For CRISPR/Cas9-mediated gene disruption, sgRNAs for SETD6 were cloned to the plentiCRISPR plasmid (Addgene #49535) as described in Chen *et al.* [8]. Following transfection and puromycin selection, single clones were isolated and expanded. For stable transfection, retroviruses were produced by transfecting HEK293T with pWZL-empty or pWZL-FLAG-SETD6 and with the plasmids encoding VSV and gag-pol. SETD6 KO HeLa cells were infected with the viral supernatants and selected with 500 μg/ml hygromycin. For synthetic peptide (synthesized at GLS-biochem in TFA, at 95% purity) introduction into cells, 24 h after plating, medium was replaced with no serum media in which the relevant peptide was added. 4 h after serum starvation, cells were washed once and fresh complete media was added for an overnight incubation.

### Peptide cloning

In order to clone the RelA302-316 peptide into Flag and GFP tag expression vectors, we generated the peptide by annealing the two primers: RelA-pep-fw 5′- CGCGCCCGCAAACGCACCTACGAGACCTTCAAAAGCATCATGAAAAAAAGCTGATTAAT-3′ and RelA-pep-rev 5′- TAATCAGCTTTTTTTCATGATGCTTTTGAAGGTCTCGTAGGTGCGTTTGCGGG-3′. The resulting fragment was then digested and cloned into the pCDNA-flag and pEGFP-C1 plasmids.

### Recombinant protein expression and purification

*Escherichia coli* BL21 transformed with a plasmid expressing a protein of interest were grown in LB media. Bacteria were harvested by centrifugation after IPTG induction at 37°C for 4 hours and lysed by sonication on ice (18% amplitude, 1 min total, 10 sec on/off). GST fusion proteins (pGex 6P1 backbone) were purified on glutathione-sepharose 4B (GE) and eluted with 10 mg/ml reduced glutathione (Sigma). His-tagged (pET-Duet1 backbone) purified proteins were purified on Ni-NTA beads (Pierce) and eluted with 500 mM imidazole buffer, followed by an over-night dialysis. Recombinant SETD6 was over-expressed and purified from insect cells as previously described [6].

### Antibodies

Antibodies used were: anti-SETD6 (Genetex, GTX629891), anti-Plk1 (Millipore, 05-844), anti-pan-methyl (Abcam, ab23366), anti-FLAG M2 (Sigma, F1804), anti-GAPDH (Abcam, G041) and anti-GST (Abcam, ab9085). HRP-conjugated secondary antibodies (goat anti-rabbit and goat anti-mouse) were purchased from Jackson ImmunoResearch (111-035-144 and 115-035-062, respectively).

### Western blot analysis, immunoprecipitation, peptide pull-down and MBT pull-down

For western blot analysis, cells were homogenized and lysed in RIPA buffer (50 mM Tris-HCl pH 8, 150 mM NaCl, 1% NP-40, 0.5% sodium deoxycholate, 0.1% SDS, 1 mM DTT and 1:100 protease inhibitor cocktail (Sigma)). Samples were heated at 95°C for 5 min in Laemmli sample buffer and resolved by SDS-PAGE, followed by western blot analysis. Immunoprecipitation was performed using protein A/G agarose beads (Santa Cruz, SC-2003) conjugated to the antibody of interest. Briefly, ∼200 μg of proteins extracted from cells using RIPA buffer were incubated overnight at 4°C with 15 μl pre-washed protein A/G beads. The beads were then washed 3 times with RIPA buffer, heated for 5 min in Laemmli sample buffer at 95°C and resolved on a 10% SDS-PAGE gel followed by western blot analysis. For peptide pull-down, similar protocol was used only using Streptavidin beads (GE, 17-5113-01) conjugated to 3 μg peptide. MBT (Malignant Brain Tumor) pull-downs were performed as previously described [18, 19].

### RNA extraction, reverse transcription and real-time qPCR

RNA extraction was performed using the NucleoSpin RNA Kit (Macherey-Nagel). Extracted RNA was reverse-transcribed to cDNA using the iScript cDNA Synthesis Kit (Bio-Rad) according to the manufacturer's instructions. Real-time PCR was carried out using the UPL probe library system (Roche). The real-time qPCR primers were the following; GAPDH; forward, 5′-AGCCACATCGCTCAGACAC-3′, reverse, 5′-GCCCAATACGACCAAATCC-3′, IL-6; forward, 5′- G ATGAGTACAAAAGTCCTGATCCA-3′, reverse, 5′- CTG CAGCCACTGGTTCTGT-3′, IL-8; forward, 5′- GAGCA CTCCATAAGGCACAAA-3′, reverse, 5′- ATGGTTCCT TCCGGTGGT-3′, TNF; forward, 5′- CGCTCCCCAAGAA GACAG-3′, reverse, 5′- AGAGGCTGAGGAACAAGCA C-3′. All samples were amplified in triplicates in a 384-well plate using the following cycling conditions: 2 min at 50°C, 10 min at 95°C, and 40 cycles at 95°C for 15 sec followed by 1 min at 60°C. The reaction was performed using 0.2 μl cDNA, 0.45 μl of each primer and LightCycler^®^ 480 Probes Master Mix for total volume reaction of 12 μl. Gene expression levels were analyzed relative to the housekeeping gene GAPDH and controls of the experiment.

### Immunofluorescence

Cells transiently expressing GFP, GFP-RelA302-316, mCherry-H2B and mCherry-SETD6 were analyzed using EVOS FL cell imaging station (ThermoFisher) without fixation. For imaging of the synthetic peptide, either a biotin or a FITC tagged peptide was introduced into the cells. For the biotin tagged peptide cells were fixed with 4% PFA, permeabilized with 0.5% Triton X-100 and blocked with 10% fetal bovine serum for 30 min. Cells were then stained with a strep conjugated Alexa647 fluorophore (Jackson). Following staining, cells were mounted on slides with the DAPI fluoromount-G mounting solution (SoutherBiotech). For the FITC tagged peptide, cells were prepared same however without permebilization and staining. Images were captured using the *Olympus FV1000 Inverted Confocal IX81* Microscope.

### Surface plasmon resonance (SPR) spectroscopy

The constants for binding of the relevant peptides to His-SETD6 was determined by SPR spectroscopy on a ProteOn XPR36 (Bio-Rad, CA, USA), as follows. His-SETD6 was immobilized on the surface of a ProteOn GLM Sensor chip using the amine coupling reagents, sulfo-NHS (0.1 M Nhydroxysuccinimide) and EDC (0.4 M 1-ethyl-3- (3-dimethylaminopropyl)-carbodiimide, Bio-Rad). The proteins (5 μg and 8 μg) were each covalently immobilized on the chip in 10 mM sodium acetate buffer, pH 4.5, to give 7052 and 9440 response units (RU) for His-SETD6 5 μg and 8 μg, respectively. BSA (5 μg, 8001 RU) was immobilized on the chip as a negative control. Unbound esters were deactivated with 1 M ethanolamine. The vp22, vp22-RelA, Antp and Antp-RelA peptides (the analytes) were then allowed to flow over the surface-bound His-SETD6, at a concentration of 1 μM and a flow rate of 100 μl/min. After regeneration, the VP22-RelA peptide was allowed to enter the system at concentrations of 62.5, 125, 250, 500 and 1000 nM and a flow rate of 100 μl/min. While the analyte was flowing over the surface (for 4 min), the interaction between His-SETD6 and vp22-RelA was determined. The next step was to examine the dissociation of the proteins, while allowing PBST to flow over the surface. After each run, a regeneration step was performed with 50 mM NaOH at a flow rate of 100 μl/min. For each protein complex, a sensorgram was generated from the RUs measured during the course of the protein-protein interaction minus the values of the BSA background channel. The dissociation constant (KD) was determined from the sensorgram of the equilibrium binding phase.

### *In vitro* methylation assay

Methylation assays were performed with recombinant proteins. Methylation reactions (total volume of 25 μl) contained 4 μg His-Plk1 and/or 4 μg GST-SETD6 and/or different peptides at indicated concentrations, 2 mCi ^3^H-labeled S-adenosyl methionine (Perkin-Elmer) and PKMT buffer (20 mM Tris-HCl pH 8, 10% glycerol, 20 mM KCl, 5 mM MgCl_2_) and were incubated overnight at 30°C. Methylation reactions were resolved by SDS-PAGE for Coomassie staining (Expedeon, Instant*Blue*) and exposed to autoradiography.

### Enzyme-linked immunosorbent assay (ELISA)

The surface of a high binding 96-well plate (Greiner Microlon) was coated with streptavidin (Pierce) at 1 μg/mL in PBS and incubated for 1 hour at room temperature. The plate was washed three times with PBST followed by the addition of a biotinylated peptide at 20 μM in PBS and incubated for 1 hour at room temperature. The plate was then washed three times with PBST and incubated with PBS+BSA 3% (PBS-BSA) for one hour at room temperature for blocking. After 3 washes with PBST, the plate was covered with 0.5 μg GST-SETD6 or GST protein (negative control) diluted in 1% BSA in PBST for 1 h. Plates were then washed and incubated with primary antibody (anti-GST, 1:4000 dilution) followed by incubation with HRP-conjugated secondary antibody (goat anti-rabbit, 1:2000 dilution) for 1 h. Finally, TMB reagent (Dako) and then 1N H_2_SO_4_ were added, and the absorbance at 450 nm was detected using a Tecan Infinite M200 plate reader.

### Wound healing assay

Transfected cells and control cells were plated separately overnight to achieve a confluent cell layer in 6-well culture plates in duplicates. The growth medium was then switched to serum-free medium for 4 h while introducing the relevant peptide, and then washed and switched back to complete media for 16 h before making a scratch on the cell layer with a tip. Images were captured (3 images per well) right before scratch was made and 24 h after it. Migration was calculated by averaging the gap during a 24 h post-scratch period.

### PrestoBlue viability assay

For viability counts, cells were plated at 25,000 cells/well in a 48-well plate, then submitted to serum starvation while a tested peptide was introduced in triplicates for 4 hours. After which, cells were washed once and fresh complete medium was added and the cells were incubated 18 hours. Following the incubation, media was removed from cells and new media containing 10% PrestoBlue (Invitrogen) was added to the cells for 20 min at 37°C. The number of viable cells was measured using a Tecan Infinite M200 plate reader by measuring absorbance at 560 and 590 nm.

## SUPPLEMENTARY MATERIALS FIGURES



## References

[1] Zhao Y, Jensen ON (2009). Modification-specific proteomics: strategies for characterization of post-translational modifications using enrichment techniques. Proteomics.

[2] Karve TM, Cheema AK (2011). Small changes huge impact: the role of protein posttranslational modifications in cellular homeostasis and disease. J Amino Acids.

[3] Seo J, Lee KJ (2004). Post-translational modifications and their biological functions: proteomic analysis and systematic approaches. J Biochem Mol Biol.

[4] Hamamoto R, Saloura V, Nakamura Y (2015). Critical roles of non-histone protein lysine methylation in human tumorigenesis. Nat Rev Cancer.

[5] Yeates TO (2002). Structures of SET domain proteins: protein lysine methyltransferases make their mark. Cell.

[6] Levy D, Kuo AJ, Chang Y, Schaefer U, Kitson C, Cheung P, Espejo A, Zee BM, Liu CL, Tangsombatvisit S, Tennen RI, Kuo AY, Tanjing S (2011). Lysine methylation of the NF-kappaB subunit RelA by SETD6 couples activity of the histone methyltransferase GLP at chromatin to tonic repression of NF-kappaB signaling. Nat Immunol.

[7] Vershinin Z, Feldman M, Chen A, Levy D (2016). PAK4 Methylation by SETD6 Promotes the Activation of the Wnt/beta-Catenin Pathway. J Biol Chem.

[8] Chen A, Feldman M, Vershinin Z, Levy D (2016). SETD6 is a negative regulator of oxidative stress response. Biochim Biophys Acta.

[9] O'Neill DJ, Williamson SC, Alkharaif D, Monteiro IC, Goudreault M, Gaughan L, Robson CN, Gingras AC, Binda O (2014). SETD6 controls the expression of estrogen-responsive genes and proliferation of breast carcinoma cells. Epigenetics.

[10] Marqus S, Pirogova E, Piva TJ (2017). Evaluation of the use of therapeutic peptides for cancer treatment. J Biomed Sci.

[11] Okada AK, Teranishi K, Lobo F, Isas JM, Xiao J, Yen K, Cohen P, Langen R (2017). The Mitochondrial-Derived Peptides, HumaninS14G and Small Humanin-like Peptide 2, Exhibit Chaperone-like Activity. Sci Rep.

[12] McBrayer DN, Tal-Gan Y (2017). Recent Advances in GLP-1 Receptor Agonists for Use in Diabetes Mellitus. Drug Dev Res.

[13] Qi GB, Zhang D, Liu FH, Qiao ZY, Wang H (2017). An “On-Site Transformation” Strategy for Treatment of Bacterial Infection. Adv Mater.

[14] Mohamed MF, Brezden A, Mohammad H, Chmielewski J, Seleem MN (2017). A short D-enantiomeric antimicrobial peptide with potent immunomodulatory and antibiofilm activity against multidrug-resistant Pseudomonas aeruginosa and Acinetobacter baumannii. Sci Rep.

[15] Shoji-Kawata S, Sumpter R, Leveno M, Campbell GR, Zou Z, Kinch L, Wilkins AD, Sun Q, Pallauf K, MacDuff D, Huerta C, Virgin HW, Helms JB (2013). Identification of a candidate therapeutic autophagy-inducing peptide. Nature.

[16] Yu X, Xu Z, Lei J, Li T, Wang Y (2015). VP22 mediates intercellular trafficking and enhances the in vitro antitumor activity of PTEN. Mol Med Rep.

[17] Elliott G, O'Hare P (1997). Intercellular trafficking and protein delivery by a herpesvirus structural protein. Cell.

[18] Carlson SM, Moore KE, Green EM, Martin GM, Gozani O (2014). Proteome-wide enrichment of proteins modified by lysine methylation. Nat Protoc.

[19] Moore KE, Carlson SM, Camp ND, Cheung P, James RG, Chua KF, Wolf-Yadlin A, Gozani O (2013). A general molecular affinity strategy for global detection and proteomic analysis of lysine methylation. Mol Cell.

[20] Chang Y, Levy D, Horton JR, Peng J, Zhang X, Gozani O, Cheng X (2011). Structural basis of SETD6-mediated regulation of the NF-kB network via methyl-lysine signaling. Nucleic Acids Res.

[21] Kanda T, Sullivan KF, Wahl GM (1998). Histone-GFP fusion protein enables sensitive analysis of chromosome dynamics in living mammalian cells. Curr Biol.

[22] Shi NQ, Qi XR, Xiang B, Zhang Y (2014). A survey on “Trojan Horse” peptides: opportunities, issues and controlled entry to “Troy”. J Control Release.

[23] Derossi D, Joliot AH, Chassaing G, Prochiantz A (1994). The third helix of the Antennapedia homeodomain translocates through biological membranes. J Biol Chem.

[24] Bolhassani A, Jafarzade BS, Mardani G (2017). *In vitro* and *in vivo* delivery of therapeutic proteins using cell penetrating peptides. Peptides.

[25] Levy D, Liu CL, Yang Z, Newman AM, Alizadeh AA, Utz PJ, Gozani O (2011). A proteomic approach for the identification of novel lysine methyltransferase substrates. Epigenetics Chromatin.

[26] Mukherjee N, Cardenas E, Bedolla R, Ghosh R (2017). SETD6 regulates NF-kappaB signaling in urothelial cell survival: Implications for bladder cancer. Oncotarget.

[27] Liu L, Kimball S, Liu H, Holowatyj A, Yang ZQ (2015). Genetic alterations of histone lysine methyltransferases and their significance in breast cancer. Oncotarget.

[28] Kanchi KL, Johnson KJ, Lu C, McLellan MD, Leiserson MD, Wendl MC, Zhang Q, Koboldt DC, Xie M, Kandoth C, McMichael JF, Wyczalkowski MA, Larson DE (2014). Integrated analysis of germline and somatic variants in ovarian cancer. Nat Commun.

[29] Nekhai S, Bottaro DP, Woldehawariat G, Spellerberg A, Petryshyn R (2000). A cell-permeable peptide inhibits activation of PKR and enhances cell proliferation. Peptides.

[30] Bonny C, Oberson A, Negri S, Sauser C, Schorderet DF (2001). Cell-permeable peptide inhibitors of JNK: novel blockers of beta-cell death. Diabetes.

[31] Fosgerau K, Hoffmann T (2015). Peptide therapeutics: current status and future directions. Drug Discov Today.

[32] Shan CM, Wang J, Xu K, Chen H, Yue JX, Andrews S, Moresco JJ, Yates JR, Nagy PL, Tong L, Jia S (2016). A histone H3K9M mutation traps histone methyltransferase Clr4 to prevent heterochromatin spreading. Elife.

[33] Jayaram H, Hoelper D, Jain SU, Cantone N, Lundgren SM, Poy F, Allis CD, Cummings R, Bellon S, Lewis PW (2016). S-adenosyl methionine is necessary for inhibition of the methyltransferase G9a by the lysine 9 to methionine mutation on histone H3. Proc Natl Acad Sci USA.

[34] Lewis PW, Muller MM, Koletsky MS, Cordero F, Lin S, Banaszynski LA, Garcia BA, Muir TW, Becher OJ, Allis CD (2013). Inhibition of PRC2 activity by a gain-of-function H3 mutation found in pediatric glioblastoma. Science.

[35] Ball KL, Lain S, Fahraeus R, Smythe C, Lane DP (1997). Cell-cycle arrest and inhibition of Cdk4 activity by small peptides based on the carboxy-terminal domain of p21WAF1. Curr Biol.

