# Supplementary material for: Peptide inhibition of the SETD6 methyltransferase catalytic activity
Source: Oncotarget. 2017 Dec 21;9(4):4875–85. doi: 10.18632/oncotarget.23591 (PMC5797019; doi:10.18632/oncotarget.23591)
Supplement: Supplementary file 1 [file oncotarget-09-4875-s001.pdf]

Peptide inhibition of the SETD6 methyltransferase catalytic activity

SUPPLEMENTARY MATERIALS

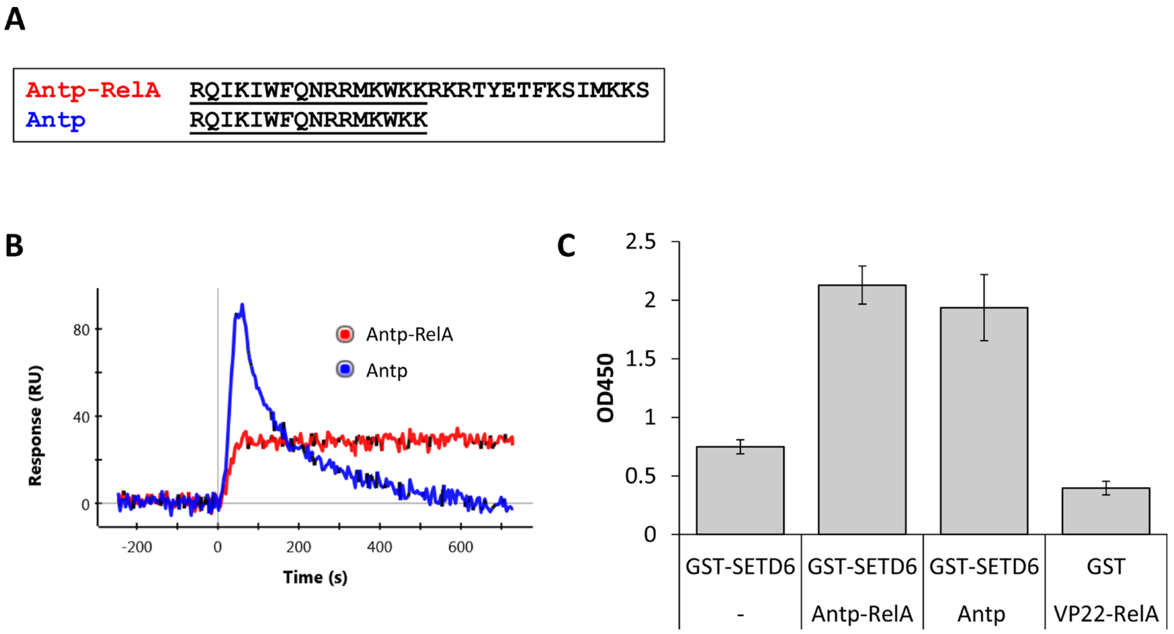

Supplementary Figure 1: The Antp-RelA peptide binds SETD6 un-specifically. (**A**) Sequence of Antp-RelA and Antp peptides. (**B**) 1 uM of the Antp-RelA and Antp peptides (analytes) were allowed to flow over the GLM chip bound His-SETD6. Binding was analyzed using SPR spectroscopy with the ProteOn XPR36 system. (**C**) ELISA-based analysis of the interaction between recombinant GST-SETD6 and the biotinylated peptides. The 96-well plate was coated with 100 µg streptavidin to which 20 µM of the indicated biotinylated peptides were bound. Then peptides were covered with 0.5 µg GST-SETD6 or GST. Signal detection was obtained using anti-GST antibody followed by secondary HRP-conjugated antibody (error bars, s.e.m.).

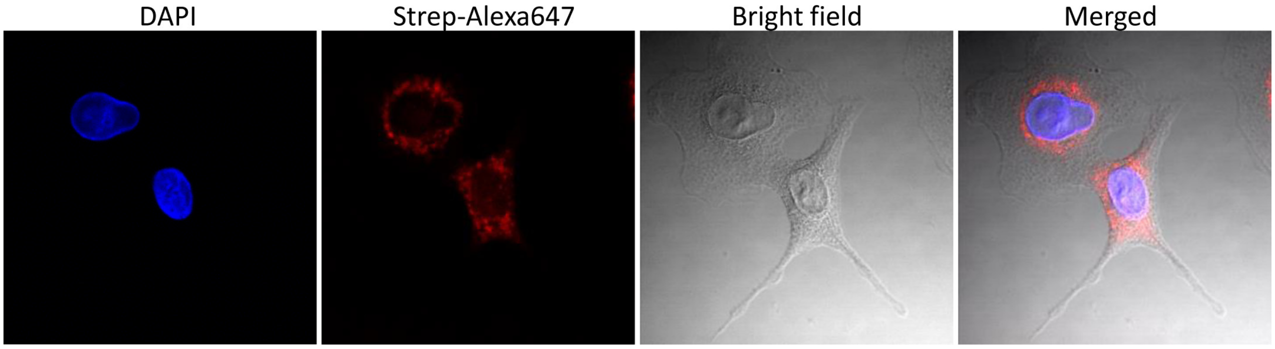

Supplementary Figure 2: The vp22-RelA peptide penetrates cells. A549 cells were treated with the biotinylated vp22-RelA peptide, then fixed and permeabilized using methanol and stained with Alexa-fluor 647 streptavidin. Then coverslips were mounted with DAPI containing mounting solution and visualized using confocal microscopy.
